# Effects of vedolizumab in Japanese patients with Crohn’s disease: a prospective, multicenter, randomized, placebo-controlled Phase 3 trial with exploratory analyses

**DOI:** 10.1007/s00535-019-01647-w

**Published:** 2019-12-13

**Authors:** Kenji Watanabe, Satoshi Motoya, Haruhiko Ogata, Takanori Kanai, Toshiyuki Matsui, Yasuo Suzuki, Mitsuhiro Shikamura, Kenkichi Sugiura, Kazunori Oda, Tetsuharu Hori, Takahiro Araki, Mamoru Watanabe, Toshifumi Hibi

**Affiliations:** 1 Department of Intestinal Inflammation Research, Hyogo College of Medicine, Hyogo, Japan; 2 IBD Center, Hokkaido Prefectural Welfare Federation of Agricultural Cooperative, Sapporo-Kosei General Hospital, Sapporo, Japan; 3 Endoscopic Center, Keio University School of Medicine, Tokyo, Japan; 4 Division of Gastroenterology and Hepatology, Department of Internal Medicine, Keio University School of Medicine, Tokyo, Japan; 5 Department of Gastroenterology, Fukuoka University Chikushi Hospital, Fukuoka, Japan; 6 Department of Internal Medicine, Toho University Medical Center Sakura Hospital, Chiba, Japan; 7 Takeda Development Center Japan, Takeda Pharmaceutical Company Limited, Osaka, Japan; 8 Regenerative Medicine Unit, Takeda Pharmaceutical Company Limited, Kanagawa, Japan; 9 Department of Gastroenterology and Hepatology, Tokyo Medical and Dental University, Tokyo, Japan; 10 Center for Advanced Inflammatory Bowel Disease Research and Treatment, Kitasato University Kitasato Institute Hospital, 5-9-1 Shirokane, Minato-ku, Tokyo 108-8462 Japan

**Keywords:** Crohn’s disease, Vedolizumab, α_4_β_7_ integrin, Biologic, Randomized controlled trial

## Abstract

**Background:**

Vedolizumab is a gut-selective humanized antibody that binds the α_4_β_7_ integrin. We evaluated efficacy and safety of vedolizumab in Japanese patients with moderate-to-severe Crohn’s disease (CD).

**Methods:**

In this Phase 3, double-blind study (NCT02038920), 157 patients were randomized to receive intravenous vedolizumab 300 mg (*n* = 79) or placebo (*n* = 78) at Weeks 0, 2, and 6 (induction phase). Patients with CD activity index (CDAI)-70 response at Week 10 were randomized to receive vedolizumab 300 mg (*n* = 12) or placebo (*n* = 12) at Week 14, then every 8 weeks until Week 54 (maintenance phase). Primary endpoints were ≥ 100-point reduction in CDAI (CDAI-100 response) at Week 10 for induction, and clinical remission (CR: CDAI ≤ 150) at Week 60 for maintenance.

**Results:**

At Week 10, 26.6% of patients who received vedolizumab and 16.7% who received placebo achieved CDAI-100 response (odds ratio [OR] [95% confidence interval (CI)] 1.80 [0.82–3.96]; *p* = 0.145). At Week 60, 41.7% of vedolizumab-treated patients and 16.7% of placebo-treated patients achieved CR (OR [95% CI] 3.57 [0.53–23.95];* p* = 0.178). The incidence of adverse events was similar in both treatment groups in both induction and maintenance phases. In patients without prior anti-TNFα exposure or with inadequate response to anti-TNFα, vedolizumab showed improved outcomes over placebo in the induction phase. Age might be a possible predictive factor of CR for future research.

**Conclusion:**

Vedolizumab showed a numerically greater efficacy versus placebo as induction therapy, but the difference was not statistically significant. Vedolizumab also showed a numerically greater efficacy in maintenance therapy, and was well tolerated.

**Electronic supplementary material:**

The online version of this article (10.1007/s00535-019-01647-w) contains supplementary material, which is available to authorized users.

## Introduction

The incidence of Crohn’s disease (CD) is lower in Asian countries than in Western ones. However, CD prevalence in Japan has rapidly increased over recent decades, from 5.9 per 100,000 people in 1991 to 30.1 per 100,000 people in 2013 [1]. As no curative treatment for CD has been established, therapeutic strategies focus on control of disease activity via induction and maintenance therapy, and measures to improve quality of life [2]. These strategies include: treatment with 5-aminosalicylic acid, immunomodulators, steroids, or biologics (e.g., anti-TNFα); surgery; and nutrition therapy [2-4]. Notably, although anti-TNFα is generally effective, there have been reports of safety concerns and primary loss or loss of response [5, 6].

Vedolizumab is a humanized immunoglobulin G1 monoclonal antibody that binds exclusively to α_4_β_7_ integrin, blocking its interaction with mucosal addressing cell adhesion molecule-1 and thereby specifically modulating gut lymphocyte trafficking [7]. In the GEMINI 2 (assessing outcomes of vedolizumab induction and maintenance therapies in patients with active CD for whom ≥ 1 prior CD therapies had failed) and GEMINI 3 trials (assessing outcomes of vedolizumab induction therapy in active CD, focusing on patients with previous anti-TNFα failure), vedolizumab demonstrated efficacy in moderate-to-severe CD, leading to wide approval for the treatment of CD [8, 9]. As Japanese patients were not included in GEMINI 2 or 3, efficacy and safety of vedolizumab in this population have not yet been evaluated.

Genetic backgrounds and phenotypes of inflammatory bowel disease differ considerably between Asian and Western patients [10, 11]; it is therefore important to determine treatment targets, outcomes, and responses in populations with different genetic backgrounds [12]. Multivariable analysis of GEMINI 2 data identified that ethnicity was a predictive factor of remission (American Indian or Alaskan Native, Asian, Black, and Native Hawaiian or other Pacific Islander vs non-Hispanic/Latino; odds ratio [OR] and 95% confidence interval [CI], 0.30 [0.12–0.75]) [13], and a post hoc analysis of data from GEMINI 2 and 3 also showed that race (non-White vs White) tended to predict rapid response to vedolizumab at Week 2 [14]. Therefore, it is meaningful to evaluate vedolizumab efficacy and associated predictive factors in patients with CD who are of different race to the more widely studied Western patient population.

Here, we evaluated efficacy, safety, pharmacokinetics and immunogenicity of vedolizumab versus placebo as induction and maintenance therapy in Japanese patients with moderate-to-severe CD. Additionally, as subgroup or post hoc exploratory analyses, we assessed factors affecting efficacy and time courses of disease activity, to contribute to the improvement of vedolizumab treatment in clinical practice.

## Methods

### Study design

This Phase 3 randomized, double-blind, placebo-controlled, parallel group study (ClinicalTrials.gov number, NCT02038920) assessed vedolizumab as induction and maintenance therapy in Japanese patients with CD at 77 centers in Japan, in accordance with the Declaration of Helsinki, International Conference on Harmonization guidelines, and relevant laws and regulations. The protocol was approved by appropriate institutional review boards. Written informed consent was obtained from all patients.

The study design is summarized in Fig. S1. In a 14-week induction phase, eligible patients were randomized 1:1 to receive placebo or vedolizumab 300 mg by intravenous infusion at Weeks 0, 2, and 6. Dynamic randomization was performed with prior anti-TNFα use (yes/no), concomitant immunomodulator use (yes/no), concomitant corticosteroid use (yes/no), and study sites as stratification factors. Vedolizumab-treated patients who achieved a reduction in CD activity index (CDAI) score of  ≥ 70 points (CDAI-70) at Week 10 were enrolled in a 46-week maintenance phase at Week 14, where they were randomized 1:1 to receive placebo or vedolizumab 300 mg. Dynamic randomization was performed with the same stratification factors as the induction phase, plus clinical remission (CR; defined as CDAI score  ≤ 150)/non-remission at Week 10. Maintenance phase treatment was administered every 8 weeks from Week 14–54.

Randomization schedules were generated by personnel designated by the sponsor and allocations were not disclosed until opening of the study drug allocation table, except to unblinded pharmacists at each site.

Patients could enter an open-label cohort and receive vedolizumab 300 mg re-induction (at Weeks 0, 2, and 6 from the initiation of the cohort), then every 8 weeks up to 94 weeks, if they had no CDAI-70 response at Week 10, had disease worsening (a  ≥ 100-point increase in CDAI score from Week 10 value and CDAI score of  ≥ 220 on two consecutive visits during the maintenance phase), received rescue treatment during the maintenance phase, or completed 60 weeks of the maintenance phase. Results of the open-label cohort are not shown in this report. The end-of-study evaluation was performed in each patient 16 weeks after the last dose of study drug.

### Patients

Eligible patients were aged 15–80 years with a diagnosis (according to the Revised Diagnostic Criteria for CD [Research Group for Intractable Inflammatory Bowel Disease Designated by the Ministry of Health, Labour and Welfare of Japan, 2012]) of ileal, colonic or ileocolonic CD ≥ 3 months prior to receipt of study treatment. Patients were required to have a CDAI score 220–450 (inclusive) at first administration of study drug and a C-reactive protein (CRP) concentration > 0.30 mg/dL at screening, or irregular-to-round shaped ulcers,  ≥ 10 aphthous lesions, longitudinal ulcers or a cobblestone appearance in the ileum or colon by conventional ileocolonoscopy or imaging test conducted at each study site within 4 months of administration of the first dose of the study drug. Treatment failure or intolerance to corticosteroids, immunomodulators (azathioprine, 6-mercaptopurine or methotrexate), or anti-TNFα, was required within 5 years before informed consent. Patients with evidence of or suspected abscess, with a history of subtotal or total colectomy, with prior small intestine resections in  ≥ 3 locations, who had received an enteral nutrient of  > 900 kcal/day or had started an enteral nutrient of  ≤ 900 kcal/day within 20 days before the first dose of study drug, or with prior diagnosis of short bowel syndrome were excluded from the study.

The target number of patients with no previous anti-TNFα use was approximately 25% of the total study population. Patients were allowed concomitant treatment with oral 5-aminosalicylic acid, probiotics, antibiotics for CD, oral corticosteroids, immunomodulators and enteral nutrients (≤ 900 kcal/day) under specific conditions. Detailed inclusion/exclusion criteria are provided in the Supplementary Appendix.

### Study endpoints and assessments

For the induction phase, the primary efficacy endpoint was the percentage of patients with a reduction in CDAI score of  ≥ 100 points from baseline (CDAI-100 response) at Week 10. Secondary efficacy endpoints were percentage of patients who achieved CR at Week 10, and change over time in CRP concentration during the induction phase in patients with baseline CRP concentration  > 0.30 mg/dL.

For the maintenance phase, the primary efficacy endpoint was the percentage of patients who achieved CR at Week 60. Secondary efficacy endpoints were the percentage of patients with a CDAI-100 response at Week 60, percentage of patients with durable remission during the maintenance phase (defined as CR at  ≥ 80% of the scheduled visits including Week 60), and percentage of patients who received concomitant oral corticosteroids at baseline and showed CR at Week 60 without corticosteroids (corticosteroid-free remission). The primary objective of the maintenance phase was to evaluate maintenance efficacy of vedolizumab by comparing numerically with GEMINI 2 results [8].

Prespecified exploratory endpoints included change over time in CDAI score in the induction phase. For primary, secondary and prespecified exploratory endpoints, subgroup analyses by prior anti-TNFα use were prespecified based on results from GEMINI 3 [9]. Inadequate response to prior anti-TNFα was defined as patients whose response was considered inadequate (determined by investigators) despite the induction therapy in the dosage described in the package insert. Loss of response to prior anti-TNFα was defined as patients who had relapse during the scheduled maintenance therapy after achieving clinical response.

The pharmacokinetic endpoint was serum vedolizumab concentration, and the immunogenicity endpoint was the appearance in serum of anti-vedolizumab antibody (AVA), including neutralizing antibody, determined by enzyme-linked immunosorbent assay and electrochemoluminescent assay, respectively [15, 16]. Blood samples were collected immediately before administration at each visit, at Weeks 2, 6, 10, 14, 22, 30, and 60 for pharmacokinetic analysis and at Weeks 0, 10, 30, 60 and 16 weeks after the last dose for immunogenicity.

Safety endpoints included treatment-emergent adverse events (TEAEs) and serious TEAEs, coded using the Medical Dictionary for Regulatory Activities version 20.0.

### Statistical methods

There was no sufficient published data for the CDAI-100 response at Week 10 in Asian patients with CD at the planning stage of this trial. Based on findings from GEMINI 3 [9], it was estimated that a sample size of 55–76 patients per treatment group in the induction phase would provide 80–90% power (significance level of 0.1 by two-sided test), to detect a statistically significant treatment difference for the primary endpoint in the induction phase. CDAI-100 response at Week 10 was estimated as 47.2% with vedolizumab and 24.6% with placebo. For the maintenance phase, sample size was not determined because detection of a statistically significant difference between treatment groups was not part of the study design.

Analyses were performed using the full analysis set, defined as randomized patients who received at least one dose of study treatment (vedolizumab/placebo) in both the induction and maintenance phase.

CDAI-100 response at Week 10 (primary induction phase endpoint) was analyzed using the Cochran–Mantel–Haenszel test, with prior anti-TNFα use as a stratification factor. A significance level of 0.1 (two-sided test) was used for CDAI-100 response at Week 10 and CR at Week 10. To better control type I error, the statistical test for CR was planned to be conducted only if CDAI-100 response was statistically significant, although *p *values would be presented in any case. For all other endpoints, *p* values < 0.05 (two-sided test) were considered significant, but not adjusted for the multiplicity of statistical tests. Although the maintenance phase was not designed to verify study endpoints statistically, each statistical test was performed for reference. CDAI-100 response and CR were considered as no response or no remission when these endpoints were missing at the time of evaluation. Statistical tests were not conducted for differences in baseline demographics and clinical characteristics between treatment groups, because patients were randomly allocated to groups, so variation of characteristics may derive by chance. Additionally, statistical tests were not conducted for subgroup analyses due to the limited number of patients. In a post hoc analysis to explore predictive factors of CDAI-100 response and CR, p values were calculated by a univariate logistic regression model with treatment, each variable, and treatment–variable interaction. Multivariate analysis using a logistic regression model was planned when > 2 factors with *p* < 0.05 were observed in the univariate analysis. All statistical analyses were performed using SAS v9.4 (SAS Institute Inc., Cary, NC, USA).

## Results

### Patients

The first patient was screened on January 28, 2014 and the last patient visit was conducted on November 16, 2017. Of 197 patients screened, 157 were enrolled into the induction phase (vedolizumab, *n* = 79; placebo *n* = 78). Mean baseline CDAI score was 303.9 ± 63.2 in the vedolizumab group and 295.0 ± 64.8 in the placebo group. In the vedolizumab group, 22.8% of patients (*n* = 18) had no prior anti-TNFα exposure, compared with 20.5% (*n* = 16) in the placebo group. Patients for whom treatment with two prior anti-TNFα had failed comprised 39.2% (*n* = 31) of those in the vedolizumab group and 41.0% (*n* = 32) in the placebo group. Steroid-dependent patients comprised 21.5% (*n* = 17) in the vedolizumab group and 16.7% (*n* = 13) in the placebo group.

In total, 42 patients were enrolled into the maintenance phase. Of these patients, 24 from the vedolizumab group of the induction phase were randomized to continue vedolizumab (*n* = 12) or switch to placebo (*n* = 12) (Fig. S2).

No marked differences were observed in baseline demographics and clinical characteristics between treatment groups in both induction and maintenance phases (Table 1, Table S1). Mean baseline CDAI score at initiation of the maintenance phase (Week 10) was 147.9 ± 89.2 in the vedolizumab group and 149.7 ± 59.9 in the placebo group.

**Table 1:** Demographics and disease characteristics at baseline of patients who entered the induction phase and maintenance phase

	Induction phase		Maintenance phase	
	Vedolizumab (*n* = 79)	Placebo (*n* = 78)	Vedolizumab (*n* = 12)	Placebo (*n* = 12)
Age, years, mean (SD)	33.9 (12.3)	32.6 (10.9)	36.7 (16.8)	35.2 (13.0)
Male, *n* (%)	51 (64.6)	52 (66.7)	6 (50.0)	9 (75.0)
BMI, kg/m^2^, mean (SD)	21.2 (4.9)	19.8 (2.6)	22.1 (6.2)	21.9 (3.7)
Duration of CD, years, mean (SD)	9.0 (6.2)	9.1 (6.5)	9.0 (4.9)	7.5 (6.6)
CRP, mg/dL, mean (SD)	2.2 (2.2)	2.9 (3.2)	2.0 (1.6)	2.4 (2.5)
CDAI score at Week 0, *n* (%)				
≤ 220	0 (0)	5 (6.4)	0 (0)	0 (0)
> 220 to ≤ 330	56 (70.9)	50 (64.1)	7 (58.3)	8 (66.7)
> 330 to ≤ 450	20 (25.3)	21 (26.9)	5 (41.7)	3 (25.0)
> 450	3 (3.8)	2 (2.6)	0 (0)	1 (8.3)
CDAI score at Week 10, *n* (%)				
≤ 150	–	–	8 (66.7)	6 (50.0)
> 150 to ≤ 220	–	–	1 (8.3)	4 (33.3)
> 220 to ≤ 330	–	–	3 (25.0)	2 (16.7)
CDAI score at Week 0, mean (SD)	303.9 (63.2)	295.0 (64.8)	319.8 (79.3)	303.3 (81.7)
CDAI score at Week 10, mean (SD)	–	–	147.9 (89.2)	149.7 (59.9)
Location of the lesion, *n* (%)				
Ileal	13 (16.5)	9 (11.5)	2 (16.7)	2 (16.7)
Colonic	11 (13.9)	19 (24.4)	5 (41.7)	1 (8.3)
Ileocolonic	55 (69.6)	50 (64.1)	5 (41.7)	9 (75.0)
Smoking classification, *n* (%)				
Never smoked	46 (58.2)	42 (53.8)	10 (83.3)	5 (41.7)
Current smoker	13 (16.5)	11 (14.1)	0 (0)	1 (8.3)
Ex-smoker	20 (25.3)	25 (32.1)	2 (16.7)	6 (50.0)
Surgical history for CD, *n* (%)	24 (30.4)	30 (38.5)	3 (25.0)	3 (25.0)
Current medical condition related to fistula, *n* (%)	7 (8.9)	12 (15.4)	0 (0.0)	1 (8.3)
Prior anti-TNFα treatment, *n* (%)				
No	18 (22.8)	16 (20.5)	4 (33.3)	5 (41.7)
Yes	61 (77.2)	62 (79.5)	8 (66.7)	7 (58.3)
Prior anti-TNFα failure, *n* (%)	60 (75.9)	61 (78.2)	8 (66.7)	7 (58.3)
Inadequate response	14 (17.7)	10 (12.8)	3 (25.0)	1 (8.3)
Loss of response	45 (57.0)	46 (59.0)	5 (41.7)	6 (50.0)
Intolerance	1 (1.3)	5 (6.4)	0 (0)	0 (0)
Number of drugs of anti-TNFα failure, *n* (%)				
None	19 (24.1)	17 (21.8)	4 (33.3)	5 (41.7)
1	29 (36.7)	29 (37.2)	2 (16.7)	5 (41.7)
2	31 (39.2)	32 (41.0)	6 (50.0)	2 (16.7)
Prior immunomodulators failure, *n* (%)	39 (49.4)	40 (51.3)	7 (58.3)	6 (50.0)
Refractory	28 (35.4)	29 (37.2)	4 (33.3)	3 (25.0)
Intolerance	11 (13.9)	11 (14.1)	3 (25.0)	3 (25.0)
Prior corticosteroids failure, *n* (%)	22 (27.8)	25 (32.1)	4 (33.3)	4 (33.3)
Resistance	5 (6.3)	6 (7.7)	0 (0.0)	2 (16.7)
Dependence	17 (21.5)	13 (16.7)	4 (33.3)	2 (16.7)
Intolerance	0 (0.0)	6 (7.7)	0 (0.0)	0 (0.0)
Worst prior treatment failure, *n* (%)				
Prior anti-TNFα failure	60 (75.9)	61 (78.2)	8 (66.7)	7 (58.3)
Prior immunomodulators failure but not anti-TNFα failure	12 (15.2)	9 (11.5)	3 (25.0)	2 (16.7)
Prior corticosteroid failure only	7 (8.9)	8 (10.3)	1 (8.3)	3 (25.0)
Concomitant medication for CD at baseline, *n* (%)				
Enteral nutrient	38 (48.1)	43 (55.1)	8 (66.7)	5 (41.7)
5-Aminosalicylic acid	64 (81.0)	59 (75.6)	8 (66.7)	11 (91.7)
OC and no immunomodulators	13 (16.5)	7 (9.0)	2 (16.7)	3 (25.0)
No OC or immunomodulators	30 (38.0)	31 (39.7)	1 (8.3)	3 (25.0)
OC and immunomodulators	9 (11.4)	11 (14.1)	3 (25.0)	0 (0.0)

*BMI* body mass index, *CD* Crohn’s disease, *CDAI* Crohn’s disease activity index, *CRP* C-reactive protein, *OC* oral corticosteroids, *SD* standard deviation, *TNFα* tumor necrosis factor α

### Efficacy outcomes: induction phase

**Fig. 1:**
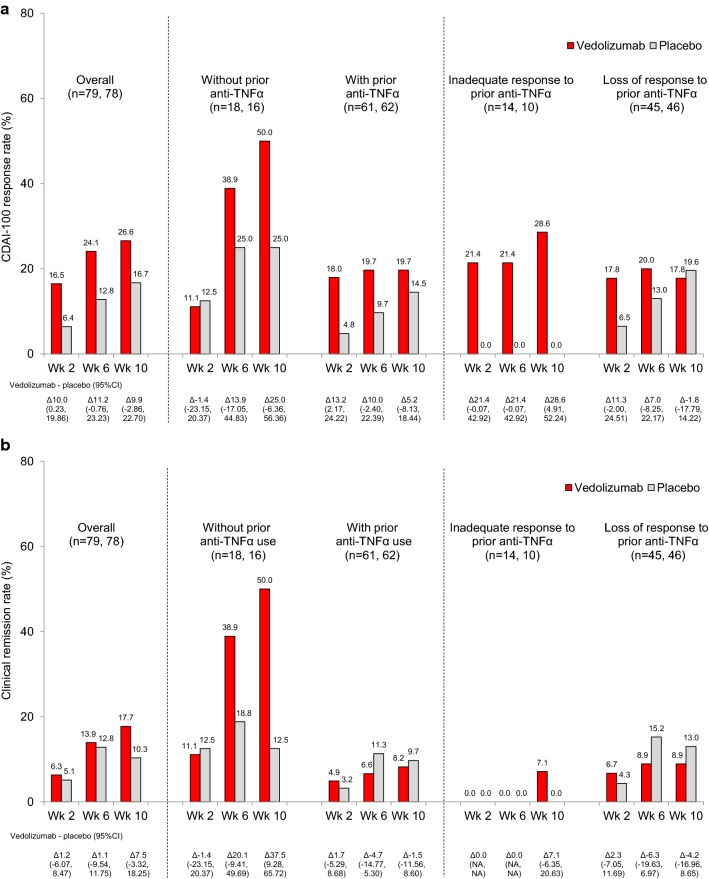
**a** CDAI-100 response and **b** clinical remission in the induction phase. *CDAI* Crohn’s disease activity index, *CI* confidence interval, *NA* not available, *TNFα* tumor necrosis factor α

CDAI-100 response and CR at Weeks 2, 6 and 10 are summarized in Fig. 1. In the induction phase, 26.6% (21/79) of patients in the vedolizumab group achieved a CDAI-100 response at Week 10 (primary endpoint) compared with 16.7% (13/78) in the placebo group; the difference was not statistically significant (adjusted OR according to prior anti-TNFα use {yes or no} [95% CI]: 1.80 [0.82–3.96]; *p* = 0.145) (Fig. 1a). In patients without prior anti-TNFα use, CDAI-100 response rate at Week 10 was numerically higher with vedolizumab (50.0%) versus placebo (25.0%). In patients with prior anti-TNFα use, CDAI-100 response rate at Week 10 was 19.7% with vedolizumab and 14.5% with placebo. In patients with inadequate response to prior anti-TNFα, CDAI-100 response rate at Week 10 was higher with vedolizumab (28.6%) versus placebo (0.0%) (95% CI 4.91–52.24).

In post hoc analysis in patients with prior anti-TNFα use, CDAI-100 response at Week 10 in ileal-type patients was inferior with vedolizumab (25.0%, 2/8) versus placebo (37.5%, 3/8), in contrast to the colonic type (66.7% [4/6] vs 18.8% [3/16]) or ileocolonic type (12.8% [6/47] vs 7.9% [3/38]) (Table S2).

CR at Week 10 was demonstrated in 17.7% (14/79) of vedolizumab-treated patients compared with 10.3% (8/78) of placebo-treated patients (adjusted OR according to prior anti-TNFα use {yes or no) [95% CI]: 1.83 [0.72–4.67]; *p* = 0.196) (Fig. 1b). Stratification by prior anti-TNFα use for CR showed similar trends to those observed for CDAI-100 response rate at Week 10; in patients without prior anti-TNFα use, CR rate at Week 10 was higher with vedolizumab (50.0%) versus placebo (12.5%) (95% CI 9.28–65.72).

### Exploratory analyses of baseline factors for remission induction at Week 10

**Fig. 2:**
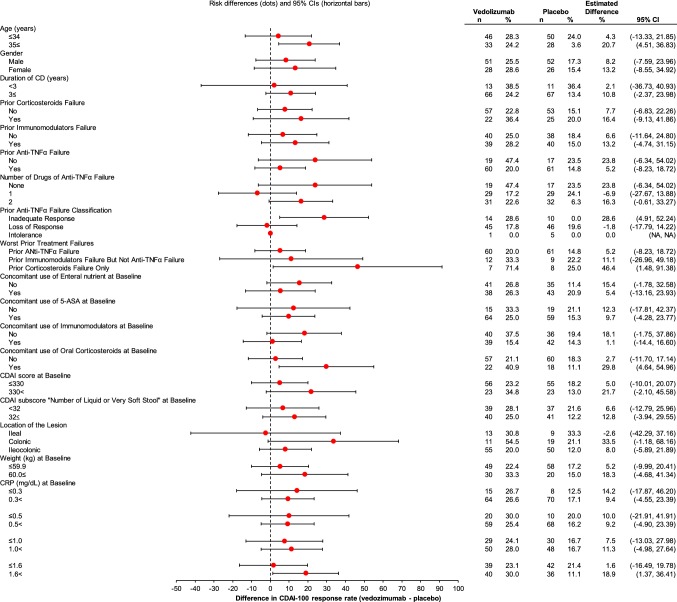
Subgroup analysis for CDAI-100 response at Week 10. *5-ASA* 5-aminosalicylic acid, *CD* Crohn’s disease, *CDAI* Crohn’s disease activity index, *CI* confidence interval, *CRP* C-reactive protein, *TNFα* tumor necrosis factor α

Subgroup analyses for CDAI-100 response and CR at Week 10 are summarized in Fig. 2 and Fig. S3, respectively. For CDAI-100 response, subgroups in which zero was not included in the 95% CI of the difference between the vedolizumab group and the placebo group were: age ≥ 35 years, inadequate response to prior anti-TNFα, prior corticosteroid failure only, concomitant use of oral corticosteroids, and CRP > 1.6 mg/dL. In all of these subgroups, vedolizumab showed a higher response or remission rate than placebo. For CR, those subgroups were: age ≥ 35 years, no prior anti-TNFα failure, and prior corticosteroid failure only.

In the post hoc univariate analysis to explore predictive factors of treatment effect, increasing age (continuous value) was the only statistically significant factor for CR (*p* = 0.04), and there were no significant factors for CDAI-100 response (Table S3).

## Changing disease activity over time: induction phase

**Fig. 3:**
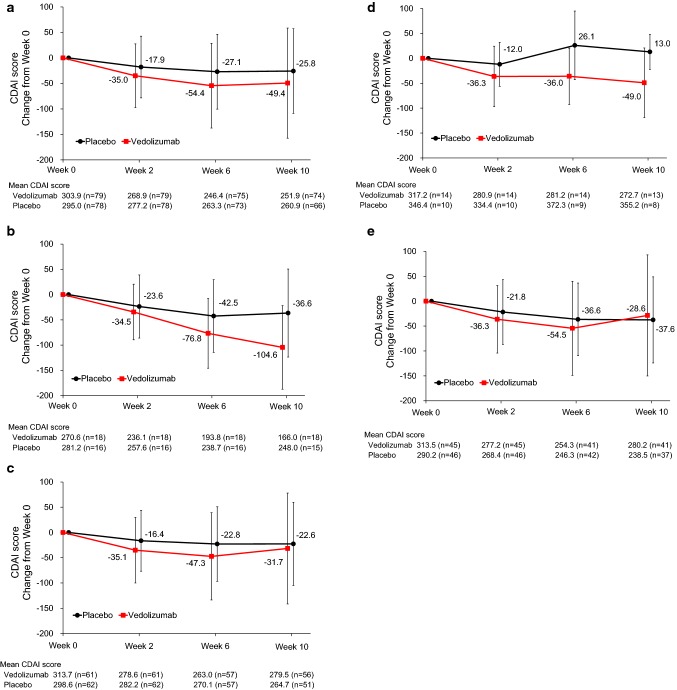
Changing CDAI score during the induction phase. **a** Overall score. **b-e** Subgroups without prior anti-TNFα use (**b**), with prior anti-TNFα use (**c**), inadequate response to prior anti-TNFα (**d**), and with loss of response to prior anti-TNFα (**e**). Data represent mean and standard deviation. *CDAI* Crohn’s disease activity index, *TNFα* tumor necrosis factor α

Mean CDAI scores decreased from baseline for both treatment groups at all measured time points during the induction phase (Fig. 3). However, the mean change from baseline in CDAI score for the vedolizumab group was greater than the placebo group at each measured time point (Fig. 3a). The difference in mean change from baseline in CDAI score between vedolizumab and placebo groups was greater in the subgroup without prior anti-TNFα use (Fig. 3b, − 104.6 ± 82.7 vs − 36.6 ± 87.2 at Week 10) than in that with prior anti-TNFα use (Fig. 3c, − 31.7 ± 109.8 vs − 22.6 ± 82.4 at Week 10). Mean CDAI score in the vedolizumab group at Week 10 was 166.0 ± 82.9 in the subgroup without prior anti-TNFα use (Fig. 3b).

Post hoc subgroup analysis showed a numerically greater difference in mean change from baseline in CDAI score between treatment groups in the subgroup with inadequate response to prior anti-TNFα (Fig. 3d, − 49.0 ± 70.0 vs  13.0 ± 35.1 at Week 10) versus the subgroup with loss of response to prior anti-TNFα (Fig. 3e,− 28.6 ± 121.5 vs − 37.6 ± 86.4 at Week 10).

Fig. S4 shows post hoc analysis of CDAI score stratified by CRP level at baseline (a cutoff of 1.6 mg/dL was used to ensure similar patients number between subgroups). In the subgroup with baseline CRP > 1.6 mg/dL, change in CDAI score at Week 10 from baseline was − 57.5 ± 116.8 in the vedolizumab group and − 5.3 ± 90.8 with placebo (Fig. S4a); no notable difference was observed in the subgroup with baseline CRP ≤ 1.6 mg/dL. In the subgroup with baseline CRP > 1.6 mg/dL, mean CDAI score in the vedolizumab group reached the lowest level at Week 6 (256.0 ± 98.2). A similar trend was observed in CDAI subscores for abdominal pain (Fig. S4b, − 19.1 ± 35.3 vs − 0.7 ± 20.4 at Week 10) and number of liquid or very soft stools (Fig. S4c, − 12.9 ± 40.2 vs − 2.1 ± 34.2).

The change over time in CRP concentration during the induction phase in patients with baseline CRP concentration > 0.30 mg/dL is shown in Fig. S5. From baseline to Week 10, median (1st quartile, 3rd quartile) CRP concentration continuously decreased by 0.295 (− 1.600, 0.550) mg/dL in the vedolizumab group (*n* = 60) and increased by 0.220 (− 0.900, 1.390) mg/dL in the placebo group (*n* = 59).

### Efficacy outcomes: maintenance phase

**Fig. 4:**
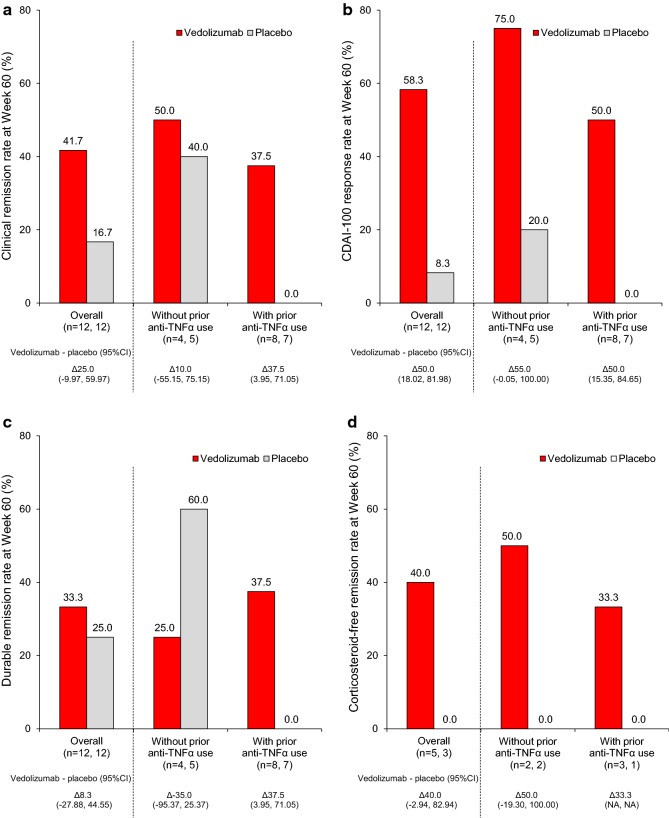
**a** Clinical remission, **b** CDAI-100 response, **c** durable remission and **d** corticosteroid-free remission in the maintenance phase. Durable remission was defined as clinical remission at ≥ 80% of the scheduled visits including Week 60. *CDAI* Crohn’s disease activity index, *CI* confidence interval, *TNFα* tumor necrosis factor α

CR at Week 60 was demonstrated in 41.7% (5/12) patients in the vedolizumab group compared with 16.7% (2/12) with placebo (OR [95% CI] 3.57 [0.53–23.95]; *p* = 0.178) (Fig. 4a). At Week 60, a significantly greater percentage of vedolizumab-treated patients achieved a CDAI-100 response (58.3% [7/12]) versus placebo (8.3% [1/12]; OR [95% CI] 15.40 [1.47–160.97]; *p* = 0.009) (Fig. 4b). Durable remission at Week 60 was demonstrated in 33.3% (4/12) patients in the vedolizumab group versus 25.0% (3/12) patients in the placebo group (OR [95% CI] 1.50 [0.25–8.84]; *p* = 0.653) (Fig. 4c). Among patients who received oral corticosteroids at baseline (*n* = 8), corticosteroid-free remission at Week 60 was 40.0% (2/5) with vedolizumab and 0.0% (0/3) with placebo (OR [95% CI] not evaluable; *p* = 0.206) (Fig. 4d). The rates of all endpoints except durable remission analyzed in the maintenance phase were numerically higher with vedolizumab versus placebo in patients without prior anti-TNFα use; no patients with prior anti-TNFα use experienced remission or response in the placebo group (difference [95% CI] 37.5% [3.95–71.0] for CR rate, and 50.0% [15.35–84.65] for CDAI-100 response rate).

### Pharmacokinetics and immunogenicity

Mean serum vedolizumab concentrations, during the induction and maintenance phase are shown in Fig. S6a and S6b, respectively. A post hoc analysis of serum vedolizumab concentrations stratified by quartile of albumin and body weight at Week 0 (factors affecting vedolizumab linear clearance [17]), is summarized in Fig. S7. Serum vedolizumab concentrations were higher in the subgroup with albumin ≥ 4.10 g/dL at Week 0 (Fig. S7a). In contrast, there were no marked differences in serum vedolizumab concentrations among the quartile groups of body weight (Fig. S7b).

**Fig. 5:**
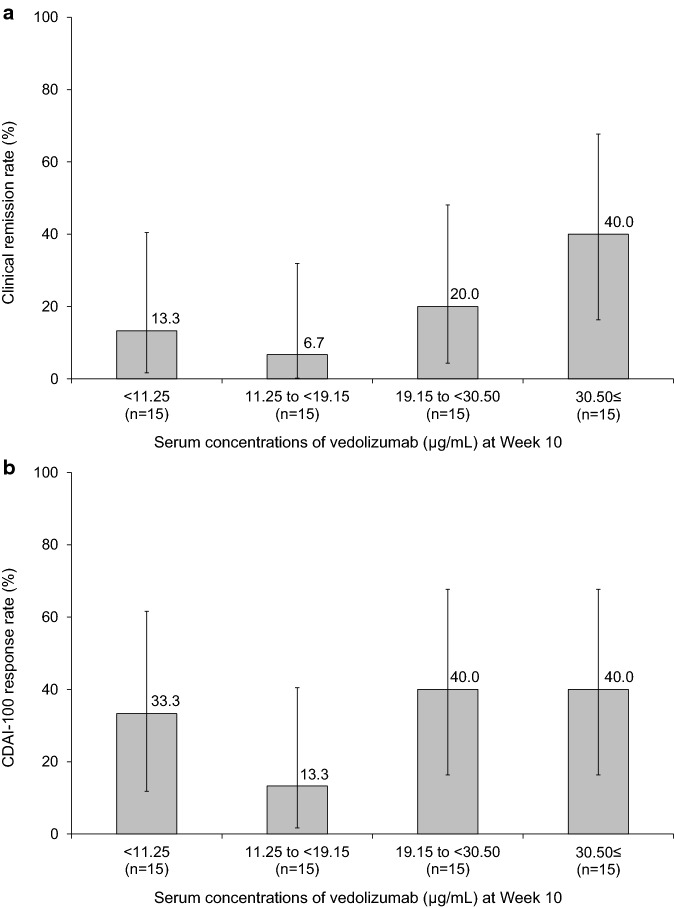
Subgroup analysis for **a** clinical remission and **b** CDAI-100 response at Week 10 by quartile serum concentrations of vedolizumab at Week 10. Bar represents 95% CI of clinical remission rate or CDAI-100 response rate. *CDAI* Crohn’s disease activity index, *CI* confidence interval

Patients who achieved a CDAI-100 response and CR at Week 10 had higher mean serum vedolizumab concentrations versus those who did not achieve these outcomes (Fig. S8). In the subgroup stratified by quartiles of serum vedolizumab concentration (Fig. 5), CR rates at Week 10 were 13.3% (2/15) in < 11.25 µg/mL, 6.7% (1/15) in 11.25 to < 19.15 µg/mL, 20.0% (3/15) in 19.15 to < 30.50 µg/mL, and 40.0% (6/15) in ≥ 30.50 µg/mL (Fig. 5a). A tendency towards higher CR rate in the group with higher serum vedolizumab concentration was observed. CDAI-100 response at Week 10 for these subgroups was 33.3% (5/15) for < 11.25 μg/mL, 13.3% (2/15) for 11.25 to < 19.15 μg/mL, 40.0% (6/15) for 19.15 to < 30.50 μg/mL, and 40.0% (6/15) for ≥ 30.50 μg/mL (Fig. 5b).

Subgroup analysis of serum vedolizumab concentrations, according to prior anti-TNFα use, showed serum vedolizumab concentrations were higher in patients without prior anti-TNFα use than with prior anti-TNFα use during both the induction phase and maintenance phase (Fig. S9).

Among 63 patients in the vedolizumab group who underwent AVA evaluation during the induction phase, one anti-TNFα failure patient was AVA-positive at any time point during the induction phase, and another was AVA-positive at baseline, prior to study drug administration. In 12 patients in the vedolizumab group who underwent AVA evaluation during the maintenance phase, no patients were AVA-positive at any time point in the induction or maintenance phase.

### Safety assessments

In the induction phase, the proportion of patients who completed the three planned infusions (Weeks 0, 2, and 6) was 93.7% with vedolizumab and 87.2% with placebo. No cases of incomplete infusion (defined as receipt of < 75% of the infusion by volume) were observed. The incidence of TEAEs with vedolizumab was similar to placebo at 62.0% and 53.8%, respectively (Table 2). For TEAEs with an incidence of ≥ 3%, no marked difference was observed between treatment groups, except exacerbation of CD, which was more frequent with placebo. Most TEAEs were mild or moderate in intensity. Serious TEAEs occurred in 8 (10.1%) vedolizumab-treated patients and 10 (12.8%) placebo-treated patients. A serious TEAE of CD exacerbation occurred in two (2.5%) patients in the vedolizumab group and 10 (12.8%) patients in the placebo group. A drug-related serious TEAE of thyroid adenoma occurred with vedolizumab. Infectious enteritis occurred in 4 (5.1%) patients during induction phase in the vedolizumab group only. Two cases were mild intensity and the other 2 cases were moderate intensity (Table S4). All 4 cases were judged by investigators not related to study drug, and recovered as the outcome. One patient in the vedolizumab group had positive findings on the objective progressive multifocal leukoencephalopathy (PML) checklist; however, this case was evaluated by the Independent Adjudication Committee and PML was ruled out.

**Table 2:** Summary of TEAEs

	Induction phase		Maintenance phase	
	Vedolizumab (*n* = 79)	Placebo (*n* = 78)	Vedolizumab (*n* = 12)	Placebo (*n* = 12)
TEAE, *n* (%)	49 (62.0)	42 (53.8)	9 (75.0)	10 (83.3)
Drug related	10 (12.7)	11 (14.1)	2 (16.7)	1 (8.3)
Intensity				
Mild	37 (46.8)	24 (30.8)	6 (50.0)	7 (58.3)
Moderate	11 (13.9)	15 (19.2)	3 (25.0)	3 (25.0)
Severe	1 (1.3)	3 (3.8)	0 (0)	0 (0)
Leading to study discontinuation	3 (3.8)	12 (15.4)	2 (16.7)	4 (33.3)
Serious TEAE, *n* (%)	8 (10.1)	10 (12.8)	2 (16.7)	4 (33.3)
Drug related	1 (1.3)	4 (5.1)	1 (8.3)	0 (0)
Leading to study discontinuation	1 (1.3)	8 (10.3)	2 (16.7)	4 (33.3)
Most common TEAEs,^a^*n* (%)				
Crohn’s disease^b^	2 (2.5)	15 (19.2)	1 (8.3)	2 (16.7)
Anal fistula	0 (0)	4 (5.1)	0 (0)	0 (0)
Pyrexia	3 (3.8)	1 (1.3)	0 (0)	1 (8.3)
Upper viral respiratory tract infection	12 (15.2)	11 (14.1)	4 (33.3)	4 (33.3)
Enteritis infectious	4 (5.1)	0 (0)	0 (0)	0 (0)
Pharyngitis	3 (3.8)	0 (0)	0 (0)	0 (0)
Headache	1 (1.3)	4 (5.1)	0 (0)	0 (0)
Hypoesthesia	4 (5.1)	0 (0)	0 (0)	0 (0)
Conjunctivitis	1 (1.3)	0 (0)	0 (0)	0 (0)
Infusion reactions,^c^*n* (%)	4 (5.1)	1 (1.3)	0 (0)	1 (8.3)
Injection site pain	1 (1.3)	0 (0)	0 (0)	0 (0)
Pyrexia	0 (0)	0 (0)	0 (0)	1 (8.3)
Infusion-related reaction	1 (1.3)	0 (0)	0 (0)	0 (0)
Body temperature increased	1 (1.3)	0 (0)	0 (0)	0 (0)
Headache	0 (0)	1 (1.3)	0 (0)	0 (0)
Throat irritation	0 (0)	1 (1.3)	0 (0)	0 (0)
Rash	1 (1.3)	0 (0)	0 (0)	0 (0)
Urticaria	1 (1.3)	0 (0)	0 (0)	0 (0)

^a^Most common TEAEs, occurring in ≥ 3% of any treatment group in the induction phase or ≥ 10% of any treatment group in the maintenance phase

^b^Exacerbation of Crohn’s disease

^c^Infusion reactions, TEAEs which occurred during the period from initiation to 1 h after completion of the study drug infusion and the investigator considered the TEAE as infusion reaction

*TEAE* treatment-emergent adverse event

In the maintenance phase, the proportion of patients who completed the planned 6 infusions (Weeks 14, 22, 30, 38, 46, and 54) was 58.3% with vedolizumab and 33.3% with placebo. No cases of incomplete infusion were observed. No marked differences in the incidence of TEAEs were observed between vedolizumab and placebo groups (75.0% vs 83.3%, respectively) (Table 2). All TEAEs in the maintenance phase were mild or moderate in severity. No patients had positive findings on either of the subjective or objective PML checklists. Drug-related serious TEAEs occurred in only one patient with vedolizumab (abdominal adhesions). No deaths occurred in the induction or maintenance phases.

## Discussion

This is the first randomized, placebo-controlled, Phase 3 study of vedolizumab in Japanese patients with moderate-to-severe CD. CDAI-100 response rate at Week 10 was numerically higher in the vedolizumab group than in the placebo group, although the difference between treatment groups was not statistically significant. The timing of the primary endpoint assessment was changed from Week 6 in GEMINI 2 and 3 to Week 10 in our study because differences in clinical outcomes between vedolizumab and placebo groups were further increased at the Week 10 timepoint in GEMINI 2 and 3 [9, 18]. In patients without prior anti-TNFα use, CDAI-100 response rates for vedolizumab and placebo at Week 10 were comparable to those in GEMINI 3 (50.0% vs 25.0% in our study; 51.0% vs 22.0% in GEMINI 3), which had a similar design with approximately three quarters of patients with prior anti-TNFα exposure, and similar baseline CDAI score to our study [9]. Furthermore, CR rate at Week 10 in our study was higher than that in GEMINI 3 (50.0% vs 12.5% in our study; 28.7% vs 13.0% in GEMINI 3). However, CDAI-100 response rate at Week 10 in patients with prior anti-TNFα use in our study was lower than that in GEMINI 3 for both treatment groups (19.7% vs 14.5% in our study; 46.8% vs 24.8% in GEMINI 3), particularly for vedolizumab. This may possibly be due to an imbalance in baseline disease localization between the treatment groups in patients with prior anti-TNFα use; the proportion of patients with colonic type was lower in the vedolizumab group than in the placebo group (9.8% vs 25.8%). Although subgroup analysis indicated higher CDAI-100 response rate at Week 10 with vedolizumab versus placebo in patients with colonic type (66.7% vs 18.8%), differences between treatment groups were lower in patients with ileal and ileocolonic type. Therefore, there was a possibility to interfere the data of efficacy by fewer patients of colonic type in the vedolizumab group. Two recent prospective trials have reported that endoscopic efficacy of vedolizumab for the ileum was inferior to that for the colon [19, 20]. However, these trials were assessed by conventional ileocolonoscopy, and there is a possibility that proximal ileal lesions can be missed with this technique [21, 22]. Furthermore, endoscopic active ileal lesions are not always successfully identified by CDAI, or by assessment of biomarkers of CD severity, for instance CRP or fecal calprotectin [23]. Further research is needed to evaluate the efficacy of vedolizumab in ileal lesions by using capsule endoscopy or balloon-assisted enteroscopy. Another possibility is the higher proportion of loss of response to prior anti-TNFα in our study versus GEMINI 3 (~ 70% vs 44%). In common with other biologics, including ustekinumab [24], efficacy of vedolizumab in CD with loss of response to anti-TNFα is lower than in bio-naïve CD. Subgroup analysis in patients with loss of response to prior anti-TNFα showed little difference in CDAI-100 response rate at Week 10 between the two treatment groups in our study. However, in patients with inadequate response to prior anti-TNFα, this response rate was higher with vedolizumab versus placebo. Loss of response to prior anti-TNFα may help to account for the lower CDAI-100 response rate at Week 10 in patients with prior anti-TNFα use in our study versus GEMINI 3. Meanwhile, in patients with inadequate response, mainly primary non-responders, to prior anti-TNFα, the inductive efficacy of vedolizumab is certainly expected, because the mode of action for anti-TNFα is different.

The data sources for our study are considered reliable because this study was conducted in compliance with good clinical practice. Therefore, we performed exploratory subgroup and post hoc analyses to evaluate factors that could contribute to the improvement of vedolizumab treatment in daily clinical practice. In the subgroups of patients aged ≥ 35 years, with inadequate response to prior anti-TNFα, prior corticosteroid failure only, concomitant use of oral corticosteroids, and CRP > 1.6 mg/dL, CDAI-100 response was superior with vedolizumab versus placebo. Similarly, in the subgroups aged ≥ 35 years, without prior anti-TNFα failure, and prior corticosteroids failure only, CR rate was superior with vedolizumab versus placebo. Moreover, increasing age may be a predictive factor for CR. But the results of the post hoc univariate analyses have a risk of false positive due to the multiplicity of 44 statistical tests, warranting careful interpretation. Vedolizumab may be more effective in patients with non-intractable CD (for instance, prior corticosteroid failure only) or older patients with CD.

CDAI-100 response rate at Week 2 was numerically higher with vedolizumab versus placebo. Evaluation of temporal changes in CDAI scores in the induction phase showed that changes from baseline were greater with vedolizumab than with placebo from Week 2 onwards. Similar results for the difference between the two treatment groups from Week 2 were also recently reported [14]. Subgroup analysis showed that CDAI-100 response rate at Week 10 was higher with vedolizumab versus placebo in all subgroups, except for those related to prior anti-TNFα use. Among subgroups with prior anti-TNFα use, the difference in mean change from baseline in CDAI score and CDAI-100 response between the vedolizumab group and the placebo group were greater in the subgroup with inadequate response to prior anti-TNFα than in that with loss of response to prior anti-TNFα. To our knowledge, no previous reports have shown changes in the CDAI score during the induction phase in CD patients with inadequate response to prior anti-TNFα. In contrast, patients with higher baseline CRP tended to show more pronounced changes in the CDAI score. In the VICTORY Consortium, patients with severe disease activity shown to be less likely to achieve CR (hazard ratio [HR] 0.54; 95% CI: 0.31–0.95) and mucosal healing (HR 0.54; 95% CI: 0.31–0.95) [25]. Therefore, vedolizumab may be more effective for remission induction in patients with moderately active CD than in those with severe active disease.

In the maintenance phase, vedolizumab treatment resulted in a numerically greater rate of CR, CDAI-100 response, durable remission and corticosteroid-free remission at Week 60 compared with placebo. A similar trend for CR was observed between our study (41.7% vs 16.7%) and GEMINI 2 (39.0% vs 21.6%). In addition, regardless of prior anti-TNFα use, the vedolizumab group exceeded the placebo group for all endpoints, except durable remission in patients without prior anti-TNFα use. The GEMINI LTS study also revealed long-term maintenance of vedolizumab efficacy regardless of prior anti-TNFα exposure [26]. In patients who achieved remission induction after administration of vedolizumab, maintenance of remission may be improved based on the lower immunogenicity of vedolizumab [27].

Vedolizumab treatment in Japanese patients with CD was well tolerated in our study, with most AEs mild to moderate in intensity. The most frequent AE in both phases was upper viral respiratory tract infection. No marked differences were reported between the vedolizumab group and the placebo group. Importantly, there were no cases of PML, a known toxicity with nonspecific α4 integrin inhibition [28]. In a recent retrospective cohort study, the number of concomitant immunosuppressive agents (corticosteroids or immunomodulators; OR, 1.72 per agent) used was associated with infections [29]. This study also reported that gastrointestinal infections were observed in the inflammatory bowel disease patients who were treated with vedolizumab (2.4 per 100 patient years of exposure). In contrast, 4 mild or moderate infectious enteritis patients were identified in our study. All 4 patients were recovered and judged by investigators not related to study drug. From the perspective for the mode of action, we should still carefully observe it as the possible specific adverse event of vedolizumab. Although it depends on the clinical course, introduction of vedolizumab as monotherapy is preferred when considering the safety profile. Furthermore, vedolizumab might be a preferred option over other immunosuppressive agents for patients who should avoid systemic immunosuppression or the elderly [30].

Pharmacokinetics and immunogenicity results in our study were consistent with those in previous studies in non-Japanese [8, 9] and Japanese patients [31, 32]. CR at Week 10 was 13.3% in the subgroup with minimum to 1st quartile of serum vedolizumab concentration, and 40.0% in the subgroup with 4th quartile to maximum of serum vedolizumab concentration. Similar trends were observed in the quartile analysis using GEMINI 3 data [33]. In general, higher concentrations of biologics provide better outcomes. Baseline albumin and body weight were reported as factors affecting vedolizumab linear clearance [17], and higher serum albumin and lower body mass at baseline demonstrated better outcomes [34]. In our analysis, vedolizumab serum concentrations were increased in the subgroup with higher albumin at Week 0, but not in the subgroup with lower body weight.

Our study is limited by the small number of patients per treatment subgroup, for instance as stratification for prior anti-TNFα use. GEMINI 2 and 3 were global trials with > 1000 or > 400 subjects, respectively [8, 9]. Our study was performed locally in Japan for a regional registration, so it was not feasible to recruit on a similar scale to the GEMINI studies. Consequently, the results of the subgroup analysis should be interpreted carefully. A further limitation is that the study was not designed to statistically evaluate efficacy during the maintenance phase; the small sample size in the maintenance phase limits the interpretation of the results. Additionally, there was no endoscopic assessment as objective monitoring.

In conclusion, vedolizumab showed numerically higher efficacy compared with placebo as induction therapy in Japanese patients with CD, but the difference was not statistically significant. Vedolizumab also showed numerically higher efficacy compared with placebo as maintenance therapy. Vedolizumab was well tolerated in this treatment setting. In patients without previous anti-TNFα exposure and those with inadequate response to anti-TNFα, vedolizumab showed improved outcomes versus placebo during the induction phase; this improvement was shown after Week 2. Age might be a possible predictive factor of remission induction for future research. Vedolizumab constitutes a new treatment option for Japanese patients with moderate-to-severe CD.

## Electronic supplementary material

Below is the link to the electronic supplementary material.

Supplementary file1 (DOCX 1322 kb)
