# Supplementary material for: Effects of vedolizumab in Japanese patients with Crohn’s disease: a prospective, multicenter, randomized, placebo-controlled Phase 3 trial with exploratory analyses
Source: J Gastroenterol. 2019 Dec 13;55(3):291–306. doi: 10.1007/s00535-019-01647-w (PMC7026209; doi:10.1007/s00535-019-01647-w)
Supplement: Supplementary file 1 — Supplementary file1 (DOCX 1322 kb) [file 535_2019_1647_MOESM1_ESM.docx]

**Inclusion Criteria**

1. In the opinion of the investigator, the patient was capable of understanding and complying with protocol requirements

2. The patient or, when applicable, the patient’s legally acceptable representative signed and dated the informed consent form prior to initiation of any study procedures

3. The patient was male or female, aged 15 to 80 years, inclusive, at the signing of informed

consent

4. A nonsterilized male patient who had a female partner of child-bearing potential had to agree to use adequate contraception during the period from the signing of informed consent to 6 months after the last dose of the study drug

5. A female patient of child-bearing potential (i.e., nonsterilized or whose last regular menses

was within previous 2 years) who had a nonsterilized male partner had to agree to use adequate contraception during the period from the signing of informed consent to 6 months after the last dose of the study drug

6. Patients who had been diagnosed with ileal, colonic or ileocolonic CD at least 3 months prior to the first dose of the study drug according to the Revised Diagnostic Criteria for Crohn's Disease issued by Research Group for Intractable Inflammatory Bowel Disease Designated by the Ministry of Health, Labor, and Welfare of Japan (2012)

7. Patients with CDAI score of 220 to 450 (inclusive) at the first dose of the study drug, and meeting at least one of the followings:

- Patients with a C-reactive protein (CRP) level of >0.30 mg/dL at the screening
- Patients with irregular-to-round shaped ulcers or multiple aphtha (≥10 lesions) in extensive area of the small or large intestine on endoscopy or imaging test within 4 months before the first dose of the study drug
- Patients with longitudinal ulcers or a cobblestone appearance in the small or large intestine on endoscopy or imaging test within 4 months before the first dose of the study drug

8. Patients whose complication of colon cancer or dysplasia was ruled out by total colonoscopy at the first dose of the study drug (or the results from total colonoscopy performed within 1 year before giving consent were available), if patients met any of the followings; patients with extensive or limited colitis of ≥8 years duration, patients aged ≥50 years, or patients with a first-degree family history of colon cancer

9. Patients who met the treatment failure criteria below with at least one of the following agents within 5 years before signing of informed consent:

- Corticosteroids
  - Resistance: patients whose response was inadequate despite the treatment of ≥40 mg/day (oral or intravenous [IV]) for ≥1 week or 30 to 40 mg/day (oral or IV) for ≥2 weeks
  - Dependence: patients who had failed to reduce the dosage to <10 mg/day due to recurrence during gradual dose reduction (oral or IV) Intolerance: patients who were unable to receive continuous treatment due to adverse reactions (e.g., Cushing's syndrome, osteopenia/osteoporosis, hyperglycaemia, insomnia, infection)
- Immunomodulators (azathioprine [AZA], 6-mercaptopurine [6-MP] or methotrexate [MTX])
  - Refractory: patients whose response was inadequate despite the treatment for ≥12 weeks
  - Intolerance: patients who were unable to receive continuous treatment due to adverse reactions (e.g., nausea/vomiting, abdominal pain, pancreatitis, liver function test abnormalities, lymphopenia, thiopurine S-methyltransferase genetic mutation, infection)
- Anti-TNFα
  - Inadequate response: patients whose response was considered inadequate (determined by investigators) despite the induction therapy in the dosage described in the package insert (this definition was different from the one used in GEMINI 2 and GEMINI 3 [Sandborn EJ et al. 2013, Sands BE et al. 2014])
  - Loss of response: patients who had relapse during the scheduled maintenance therapy after achieving clinical response (those who withdrew for other reasons than relapse were not applicable here)
  - Intolerance: patients who were unable to receive continuous treatment due to adverse reactions (e.g., infusion-related reaction, demyelinating disease, congestive heart failure, infection)

**Exclusion Criteria**

1. Patients who had an evidence of or suspected abscess

2. Patients who had a history of subtotal or total colectomy

3. Patients who had a history of small intestine resections in at least 3 locations, or a history of diagnosis of short bowel syndrome

4. Patients who had ileostomy, colostomy, internal fistula, or severe intestinal stenosis

5. Patients who had a treatment history with natalizumab, efalizumab or rituximab

6. Patients who started oral 5-ASAs, probiotics, antibiotics for CD treatment, or oral corticosteroids (≤30 mg/day) within 13 days before the first dose of the study drug. Or patients who changed dosage of or discontinued these drugs within 13 days before the first dose of the study drug if the patient had used these drugs for more than 14 days before the first dose of the study drug

7. Patients who had received 5-ASA, corticosteroid enemas/suppositories, corticosteroid IV infusion, oral corticosteroid at >30 mg/day, drugs for diarrhea-predominant irritable bowel syndrome, or Chinese herbal medicine for the CD treatment (e.g., Daikenchuto) within 13 days before the first dose of the study drug

8. Patients who had received AZA, 6-MP, or MTX within 27 days before the first dose of the study drug. However, this did not apply to patients who had used these drugs for more than 83 days before the first dose of the study drug and continued the steady dose of the drugs for more than 27 days before the first dose of the study drug

9. Patients who had received cyclosporine, tacrolimus, tofacitinib or any study drugs of low molecular compound for CD treatment within 27 days before the first dose of the study drug

10. Patients who had received adalimumab within 27 days before the first dose of the study drug or any biologics other than adalimumab within 55 days before the first dose of the study drug. However, this did not apply to patients who had received localized injections of these drugs (e.g., intraocular injection for treatment of age-related macular degeneration)

11. Patients who had received any live-vaccinations within 27 days before the first dose of the study drug

12. Patients who had undergone an enterectomy within 27 days before the first dose of the study drug or those who anticipated an enterectomy during the study

13. Patients who had received leukocytapheresis or granulocyte apheresis within 27 days before the first dose of the study drug

14. Patients who had received central venous nutrition therapy or enteral total nutrition therapy, or fasted within 20 days before the first dose of the study drug

15. Patients who had received an enteral nutrient of >900 kcal/day or who had started an enteral nutrient of ≤900 kcal/day within 20 days before the first dose of the study drug. Or patients who changed dosage of or discontinued the enteral nutrient within 20 days before the first dose of the study drug if the patient had received an enteral nutrient of ≤900 kcal/day more than 21 days before the first dose of the study drug

16. Patients who had been infected with *Clostridium difficile*, cytomegalovirus, or any other intestinal pathogen within 27 days before the first dose of the study drug

17. Patients who had an evidence of adenomatous colonic polyps that needed to be removed at the first dose of the study drug

18. Patients who had a history or a complication of large or small intestinal dysplasia

19. Patients who were suspected to have enteritis other than CD

20. Patients who were hepatitis B surface (HBs) antigen-positive or hepatitis C virus (HCV) antibody-positive at the screening. Or patients who were hepatitis B core (HBc) antibody-positive or HBs antibody positive, even though HBs antigen-negative. However, this did not apply to patients who were only HBs antibody-positive due to hepatitis B virus (HBV) vaccination, HBV-DNA-negative, HCV antigen-negative, or HCV-RNA-negative

21. Patients who had an evidence of history of tuberculosis or a suspected history of tuberculosis (including those who had findings suggesting previous tuberculosis on chest imaging procedure at the screening). However, this did not apply to patients who had completed prophylactic isoniazid, or patients who had been receiving prophylactic isoniazid for more than 21 days before the first dose of the study drug (in the latter case, the screening period were allowed to extend up to 28 days to ensure at least 21-day prophylactic isoniazid and then the study treatment was allowed to start)

22. Patients who had positive T-SPOT test or QuantiFERON test at the screening

23. Patients who had a history or complication of identified congenital or acquired immunodeficiency syndrome (e.g., not-classifiable immunodeficiency, human immunodeficiency virus [HIV] infection or organ transplantation)

24. Patients who had been affected by extraintestinal infection (e.g., pneumonia, sepsis, active hepatitis or pyelonephritis) within 27 days before the first dose of the study drug

25. Patients who had a treatment history with MLN0002

26. Female patients who were lactating at the screening, or had positive urine pregnancy test either at the screening or baseline.

27. Patients who had serious complications in the heart, lung, liver, kidney, metabolism, gastrointestinal system, urinary system, endocrine system or blood

28. Patients who had a history of a surgery requiring general anesthesia within 27 days before the first dose of the study drug, or with a schedule of a surgery requiring hospitalization during the study period

29. Patients who had a complication or a history of malignancy. However, this did not apply to the following patients:

- Patients who had a curative resection of localized skin basal cell carcinoma or had completed curative radiotherapy
- Patients who had not experienced recurrence for more than 1 year since completion of a curative resection or curative radiotherapy for skin squamous cell carcinoma
- Patients who had not experienced recurrence for more than 3 years since completion of a curative resection or curative radiotherapy for intraepithelial carcinoma of uterine cervix

For patients who had a substantially distant history of malignancy (e.g., 10 years or longer without recurrence since treatment completion), the investigator and the sponsor were to have a discussion to decide eligibility on the basis of type of malignancy and treatment applied.

30. Patients who had a history or a complication of the central nervous disorder, including stroke, multiple sclerosis, brain tumor, or neurodegenerative disease 31. Patients who had any subjective symptoms in the subjective PML checklist at the screening or baseline

32. Patients who had any of the following laboratory abnormalities at the screening;

- Hemoglobin ≤8 g/dL
- White blood cells ≤3,000/μL
- Lymphocytes ≤500/μL
- Platelets ≤100,000/μL or ≥1,200,000/μL
- Alanine aminotransferase or aspartate aminotransferase ≥3×upper limit of normal (ULN)
- Alkaline phosphatase ≥3×ULN
- Creatinine ≥2×ULN

33. Patients who had a history or a complication of alcohol dependence or illicit drug use within one year before the first dose of the study drug

34. Patients who had a history or a complication of psychotic disorder that could obstruct compliance with the study procedures

**Table S1** Demographics and disease characteristics at baseline of patients who entered the induction phase and maintenance phase with post-hoc　statistical analyses

CONSORT 2010 Explanation and Elaboration (BMJ 2010;340:c869.) didn’t recommend to perform the statistical analyses for the baseline characteristics in the randomized controlled trial. Therefore, we added this table as supplementary table.

**Table S2** Subgroup analysis for CDAI-100 response by the location of the lesion at Week 10 in patients with prior anti-TNFα use

| Item | Category | | Treatment | n | | CDAI-100 response  (n [%]) |
| --- | --- | --- | --- | --- | --- | --- |
|  |  |  |  |  |  |  |
|  |  |  |  |  |  |  |
| Location of the lesion | | Ileal | Vedolizumab Placebo | | 8 8 | 2 (25.0) 3 (37.5) |
|  |  | Colonic | Vedolizumab Placebo | | 6 16 | 4 (66.7) 3 (18.8) |
|  |  | Ileocolonic | Vedolizumab Placebo | | 47 38 | 6 (12.8) 3 (7.9) |

*CDAI* Crohn’s disease activity index, *TNFα* tumor necrosis factor α

**Table S3** Univariate analysis to explore predictive factors of CDAI-100 response and clinical remission at Week 10

| Variable | P-value for interaction of treatment and the variable | |
| --- | --- | --- |
|  | CDAI-100 Response at Week 10 | Clinical Remission at Week 10 |
| Age (years) (continuous value) | 0.14 | 0.04 |
| Age (years) (<=34 vs 35<=) | 0.10 | NA |
| Gender (Male vs Female) | 0.72 | 0.28 |
| Duration of CD (years) (continuous value) | 0.44 | 0.41 |
| Duration of CD (years) (<3 vs 3<=) | 0.51 | 0.56 |
| Prior Corticosteroids Failure (No vs Yes) | 0.70 | 0.57 |
| Prior Immunomodulators Failure (No vs Yes) | 0.61 | 0.93 |
| Prior Anti-TNFα Failure (No vs Yes) | 0.42 | 0.05 |
| Concomitant use of Enteral Nutrient at Baseline (No vs Yes) | 0.37 | 0.60 |
| Concomitant use of 5-ASA at Baseline (No vs Yes) | 0.99 | 0.90 |
| Concomitant use of Immunomodulators at Baseline (No vs Yes) | 0.32 | 0.74 |
| Concomitant use of Oral Corticosteroids at Baseline (No vs Yes) | 0.12 | 0.50 |
| CDAI Score at Baseline (continuous value) | 0.21 | 0.55 |
| CDAI Score at Baseline (<=330 vs 330<) | 0.28 | 0.93 |
| Location of the lesion (Ileal vs Colonic vs Ileocolonic) | 0.42 | 0.74 |
| Weight (kg) at Baseline (continuous value) | 0.63 | 0.79 |
| Weight (kg) at Baseline (<=59.9 vs 60.0<=) | 0.42 | 0.54 |
| CRP (mg/dL) at Baseline (continuous value) | 0.70 | 0.51 |
| CRP (mg/dL) at Baseline (<=0.3 vs 0.3<) | 0.77 | 0.96 |
| CRP (mg/dL) at Baseline (<=0.5 vs 0.5<) | 0.98 | 0.62 |
| CRP (mg/dL) at Baseline (<=1.0 vs 1.0<) | 0.81 | 0.60 |
| CRP (mg/dL) at Baseline (<=1.6 vs 1.6<) | 0.17 | 0.34 |

P-values were calculated by a logistic regression model with treatment, each variable and treatment*variable interaction.

P <0.05 is considered statistically significant. Shown are p values without adjustment for multiplicity of tests.

No multivariate analysis was performed, as because the analysis was planned to be conducted when more than two variables had with p<0.05 in the univariate analysis.

*5-ASA* 5-aminosalicylic acid, *CD* Crohn’s disease, *CDAI* Crohn’s disease activity index, *CRP* C-reactive protein, *NA* not available, *TNFα* tumor necrosis factor α

**Table S4 The cases of infectious enteritis in the vedolizumab group**

|  | AE Intensity | AE Related to Study Drug | Outcome |
| --- | --- | --- | --- |
| 1 | Moderate | No | Recovered/Resolved |
| 2 | Mild | No | Recovered/Resolved |
| 3 | Mild | No | Recovered/Resolved |
| 4 | Moderate | No | Recovered/Resolved |

**Fig. S1** Study design


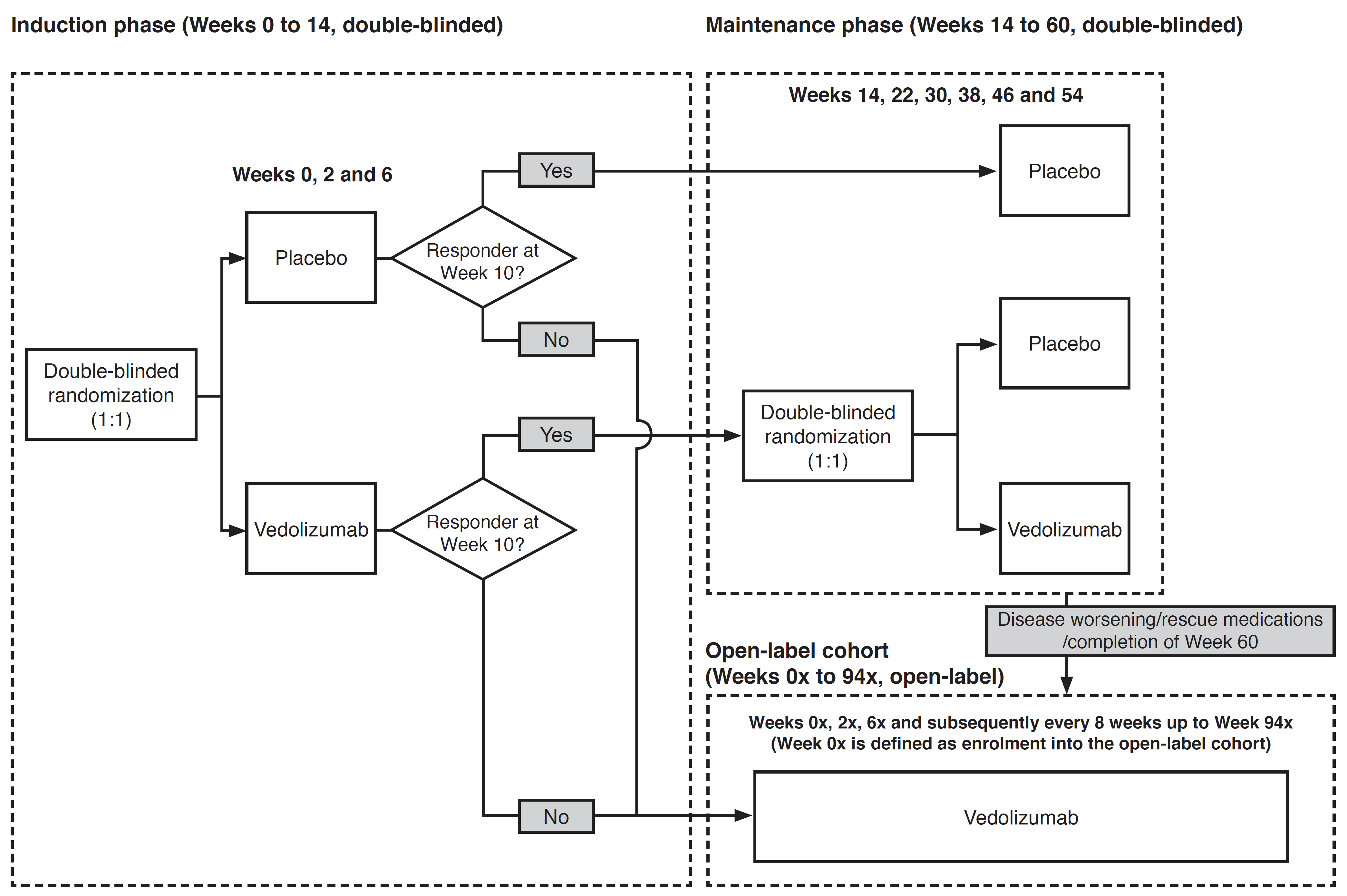


**Fig. S2** Patient disposition


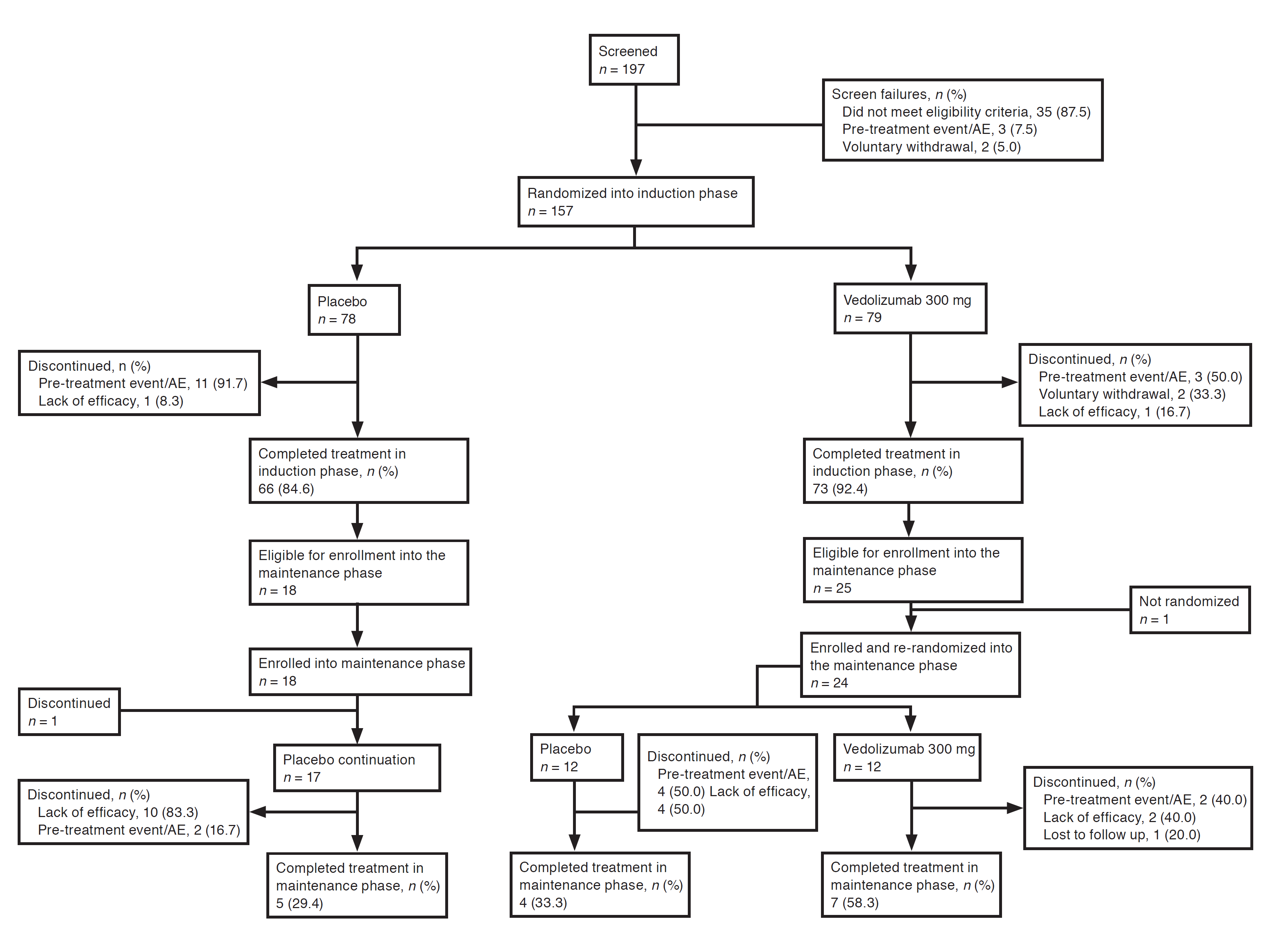


*AE* adverse event

**Fig. S3** Subgroup analysis for clinical remission at Week 10

*5-ASA* 5-aminosalicylic acid, *CD* Crohn’s disease, *CDAI* Crohn’s disease activity index, *CI* confidence interval, *CRP* C-reactive protein, *TNFα* tumor necrosis factor α

**Fig. S4** Changing CDAI score and its subscores during the induction phase stratified by CRP level at Week 0. **a** CDAI score, CDAI subscores for **b** abdominal pain, and **c** number of liquid or very soft stools.

**a**

**b**

**c**

Data represent mean and standard deviation. *CDAI* Crohn’s disease activity index, *CRP* C-reactive protein

**Fig. S5** CRP concentration during the induction phase in patients with baseline CRP concentration >0.30 mg/dL

Median CRP (mg/dL)

Vedolizumab 2.225 (n=64) 1.925 (n=64) 1.470 (n=61) 1.440 (n=60)

Placebo 2.040 (n=70) 2.030 (n=70) 1.880 (n=65) 2.160 (n=59)

Data represent median and interquartile range. *CRP* C-reactive protein

**Fig. S6** Changing serum concentrations of vedolizumab during **a** induction and **b** maintenance phase

**a**

** b**

Data represent mean and standard deviation.

**Fig. S7** Changing serum concentrations of vedolizumab stratidfed by **a** albumin and **b** body weight at Week 0

**a**

**b**

Data represent mean and standard deviation.

**Fig. S8** Subgroup analysis for serum concentrations of vedolizumab by major endpoints: **a** induction phase and **b** maintenance phase.

**a**

**b**

Data represent mean and standard deviation.

**Fig. S9** Changing serum concentrations of vedolizumab stratified by prior use of anti-TNFα during **a** induction phase and **b** maintenance phase.

**a**

**b**
